# Hypoxia and risk preferences: Mild hypoxia impacts choices for low-probability high-payoff bets

**DOI:** 10.3389/fphys.2022.960773

**Published:** 2022-08-29

**Authors:** Stefania Pighin, Nicolao Bonini, Constantinos Hadjichristidis, Federico Schena, Roberto Modena, Lucia Savadori

**Affiliations:** ^1^ Center for Mind/Brain Sciences (CIMeC), University of Trento, Rovereto, Italy; ^2^ Department of Economics and Management, University of Trento, Trento, Italy; ^3^ Research Center Sport, Mountain and Health (CERISM), University of Verona, Rovereto, Italy

**Keywords:** mild hypoxia, acute stress, decision-making, risk-taking, risky choices, gains, losses

## Abstract

Mild degrees of hypoxia are known to exert a detrimental effect on cognitive functions. In a lab study, we assessed the effect of mild hypoxia on risk-taking behavior. Participants (*N* = 25) were presented with pairs of bets of equal expected monetary value, one having a higher probability of winning/losing a lower payoff (safer bet) and one having a lower probability of winning/losing a higher payoff (riskier bet). We systematically varied the ratio of the probabilities (and corresponding payoffs) of the two bets and examined how this affected participants’ choice between them. Following a familiarization session, participants performed the task twice: once in a normoxic environment (20.9% oxygen concentration) and once in a mildly hypoxic environment (14.1% oxygen concentration). Participants were not told and could not guess which environment they were in. We found a higher preference for the riskier bet in the mild hypoxic than normoxic environment but only in the loss domain. Furthermore, as the probability ratio increased, mild hypoxia increased the preference for the riskier bet in the domain of losses but decreased it for gains. The present findings support that mild hypoxia promotes riskier choices in the loss domain and provide new insights into the impact of mild hypoxia in moderating the effect of probability ratio on risky choices.

## 1 Introduction

An adequate oxygen supply is crucial for correct cognitive functioning, and even mild degrees of hypoxia can alter cognitive abilities, including vision, attention, and memory (Legg et al., 2012; Petrassi et al., 2012; Steinman et al., 2019; Shaw et al., 2021).

However, little is known about the effects of mild hypoxia on higher-order cognitive abilities such as decision making and reasoning (e.g., Green and Morgan, 1985; Legg et al., 2014). Although the impact of mild hypoxia is not particularly striking in field studies and/or after acclimatization (Niedermeier et al., 2017; Falla et al., 2021), laboratory findings are in line with those concerning the effect of acute stressors on decisions (for a review, see Morgado et al. 2015; Starcke and Brand, 2016). They suggest that a mild oxygen depletion (equal to what is experienced at an altitude of 3,000m/9,842 ft) promotes risk-taking behavior (see also Pighin et al., 2020). Specifically, mild oxygen depletion reduced people’s tendency to prefer avoiding losses to acquiring equivalent gains (Pighin et al., 2014), and increased preference for a risky option (e.g., a 20% chance of losing €25) over a sure option of equal expected value (e.g., a 100% chance of losing €5), especially when facing potential losses (Pighin et al., 2012).

The aim of the present research was twofold. First, we aimed to investigate how mild hypoxia affects choices between pairs of risky options that differ on two dimensions (probability and payoff) but are matched in expected monetary value. Specifically, we considered pairs of bets wherein one bet posed a smaller risk by featuring a higher probability of winning (or losing) a more modest amount (hereafter, safer bet) and another bet that posed a bigger risk by featuring a lower probability of winning (or losing) a larger amount (hereafter, riskier bet). Based on previous research (Pighin et al., 2012), we expected an increased preference for the riskier bet under mild hypoxic than normoxic conditions, mainly when facing potential losses.

Second, going beyond previous research, we aimed to explore how mild hypoxia alters the preference for riskier bets as the probability ratio (i.e., the probability of the safer bet divided by the probability of the riskier bet) is systematically varied. Note that since the expected value of the bet was kept constant, varying the probability necessarily implies also varying the payoff. As it can be seen in the examples below, as the probability ratio increases, the difference between the two bets is accentuated. When the probability ratio is high, pairs include a bet with a high probability of winning (or losing) a small amount and a bet with a low probability of winning (or losing) a large amount. In contrast, when the probability ratio is low, the difference between the two bets is small. Pairs include either two high-probability low-payoff bets or two low-probability high-payoff bets.

For instance, the following pairs of bets include one safer and one riskier bet. All bets have the same expected value (EV = -2.7 euros), but the probability ratio increases as we move from A (1.13) to B (2.25) to C (9):

A. 90% chance of losing €3 vs 80% chance of losing €3.4.

B. 90% chance of losing €3 vs 40% chance of losing €6.8.

C. 90% chance of losing €3 vs 10% chance of losing €27.

The same is true for the following pairs of bets but in the domain of gains.

D. 90% chance of winning €3 vs 80% chance of winning €3.4.

E. 90% chance of winning €3 vs 40% chance of winning €6.8.

F. 90% chance of winning €3 vs 10% chance of winning €27.

In pair C, the preference for the 90% chance of losing €3 rather than for the 10% chance of losing €27 is supposed to be driven by the fear to incur a substantial loss; while in pair F, the preference for the 10% chance of winning €27 rather than the 90% chance of winning €3 is supposed to be driven by the hope of obtaining a substantial gain (Kahneman, 2012). However, this emotional trigger is virtually absent when the probability ratio is lower, as is the case for the bets in A and D. Accordingly, the proportion of choices for the riskier bet might change as a function of the probability ratio. In the current study, we examined the impact of mild hypoxia in moderating the effect of probability ratio on risky choices.

## 2 Materials and methods

### 2.1 Participants

The minimum sample size was estimated through a simulation approach (Brysbaert, 2019) implemented in R. The *a priori* power analysis suggested that a sample of 25 participants provides 80% power for detecting a very small effect size (log odds ratio = 0.18, corresponding to Cohen’s *d* = 0.1 according to Sánchez-Meca et al., 2003) for the effect of the experimental session. Accordingly, a sample of 25 right-handed university students (13 females; mean age 22 years ±2.4, ranging from 19 to 27) was involved. The study and the informed consent procedure were approved by the Research Ethics Committee of the University of Verona (Verona, Italy, Prot. N. 58). Individuals with a history of heart conditions (e.g., cardiovascular disease, angina, heart attack, etc.) were excluded because mild hypoxia can have detrimental effects on heart functioning (West et al., 2012). Participants could enroll if they provided written consent and passed a health assessment test conducted by an experienced physician.

### 2.2 Procedure

Participants were tested individually in three consecutive sessions separated by a 7-day interval: a familiarization session, a normoxic session, and a mild hypoxic session. In the familiarization and normoxic sessions, participants were exposed to an oxygen concentration of 20.9% (i.e., equivalent to an altitude of 0m/0 ft), whereas in the mild hypoxic session, the oxygen concentration was 14.1% (i.e., equivalent to an altitude of 3,000m/9,842 ft). While the familiarization session was always first, the order of the normoxic and mild hypoxic sessions was counterbalanced among participants. Both participants and experimenters were blind as to the order of the sessions. Before the beginning of the study, participants were told that “In this study, you may find yourself in a mildly hypoxic environment (simulating an altitude of 3,000 m above sea level) in some, all or none of the sessions” and no further information was provided about the oxygen condition of the individual sessions. At the beginning of each session, participants were asked to watch a 20 min documentary to allow enough time for the physiological alterations induced by mild hypoxia to occur. Then, participants were asked to perform the decision-making task (see description below). At the end of each session, participants were asked to indicate which session (normoxic or mild hypoxic) they believed they were in. The technical equipment used to measure physiological parameters was applied at the beginning of each session. Heart rate was recorded in 5 s intervals (Polar Electro Oy, Kempele, Finland), while SaO2 was measured by a portable pulse oximeter (Intermed SAT-500) placed on the index finger of the right hand at three points during a session: at the end of the video clip (about 25 min after entering in the test room) midway through the decision-making task (about 35 min after entering in the test room), and at the end of the decision-making task (about 45 min after entering in the test room).

### 2.3 Decision-making task

**FIGURE 1:**
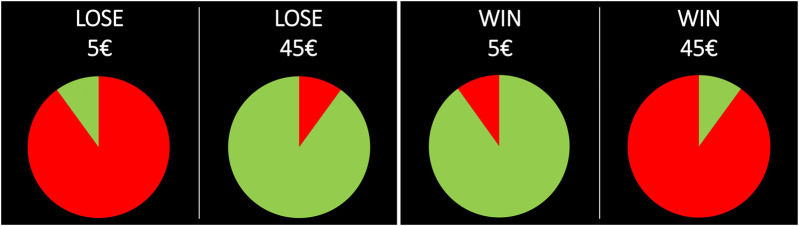
Examples of pairs of bets used in the decision-making task in the loss domain (on the left) and in the gain domain (on the right) with the probability of losing (or not winning) in red and the probability of winning (or not losing) in green.

Participants were presented with 216 pairs of bets, 108 involving potential gains and 108 potential losses. Every pair comprised two bets of the same expected value; one had a higher chance of winning (or losing) a modest amount of money (e.g., a 90% chance of winning 3€; the safer bet) while the other a smaller chance of winning (or losing) a larger amount (e.g., a 10% chance of winning 27€; the riskier bet). The probabilities of wins/losses were presented by means of a pie chart by colouring in green the relevant area and leaving the rest in red, and the payoffs were presented on the top of the pie chart (e.g., “LOSE 5€” and “LOSE 45€”; “WIN 5€” and “WIN 45€”), as it can be seen in Figure 1.

At the beginning of the study, participants were provided with an endowment of 45 euros (which covered any possible losses) that was held by the experimenter until the end of the study. The task began with an instruction phase, followed by 10 practice trials. The task was divided into four blocks of 54 randomly ordered trials each. Pairs of bets were presented for 5 s. The presentation order of the two bets was fully balanced across blocks. For each pair, participants chose one bet to play. Participants were instructed to be fast but careful. No feedback was provided after each choice, but participants were told that one bet would be extracted at random, played, and then honoured with real money at the end of three sessions. The outcome of the bet was either added to or subtracted from the endowment of 45 euros. Participants were informed that, at best, the initial endowment could be doubled (i.e., 90 euros) and that, at worst, it could be wiped out (i.e. to 0 euros).”

The protocol was programmed in E-Prime (Psychology Software Tools, Pittsburgh). Pairs were generated by starting from a set of safer bets with three different payoffs (1, 3 and 5 euros) and eight different probability levels (.2, .3, .4, .5, .6, .7, .8, and .9). For each safer bet, riskier bets were generated in order to obtain all possible probability matching between safer and riskier bets (for the complete set of pairs, see https://osf.io/x3j2q/). The probability ratio, calculated as the ratio between the probability of the safer and riskier bet, was systematically varied across pairs of bets and ranged from 1.13 (i.e., .9/.8) to 9 (i.e., .9/.1). The probability ratio was not correlated with expected value (*r* = .061). Importantly, since the two bets in a pair had the same expected monetary value (which is calculated by multiplying the probability with the payoff), the payoff ratio (i.e., the ratio between the payoff of the safer bet and that of the riskier bet) also varied across pairs and perfectly mirrored the probability ratio. Payoffs were expressed in euros and cents and ranged from 1 euro to 45 euros. Expected values ranged from 20 cents to 4.5 euros.

## 3 Results

### 3.1 Manipulation check

Oxygen depletion significantly altered physiological responses as showed by paired-sample *t*-test: in the mild hypoxic session participants’ heart rate was significantly higher (*M*_hypoxia_ = 83.4, *SD* = 11.5 vs *M*_normoxia_ = 79.6, *SD* = 11.19; *t* (24) = 3.16, *p* = .004) and their SaO2 levels significantly lower (*M*_hypoxia_ = 87.8, *SD* = 3.6 vs *M*_normoxia_ = 97.9, *SD* = .6; *t* (24) = 15.09, *p* < .001) than in the normoxic session. However, participants appeared to be generally unaware of the oxygen manipulation: of the 25 participants, 9 guessed correctly both the mild hypoxic and the normoxic condition, 6 guessed correctly the mild hypoxic condition only, 6 guessed correctly the normoxic condition only, and 4 always guessed wrong. This means that 40% of the time, participants guessed wrong and that their capacity to differentiate between the normoxic and hypoxic session did not deviate significantly from chance as showed by a McNemar’s test (*p* > .05).

### 3.2 Decision-making task

Data analysis was performed by using R, version 4.2.0 (http://www.r-project.org), with “lme4” (Bates et al., 2015) and “lsmeans” (Lenth, 2016) R packages. Scripts and outputs are available at https://osf.io/x3j2q/.

Participants’ decisions were analyzed by means of a binomial generalized linear mixed model (GLMM) *via* maximum likelihood estimation method with the “glmer” function (Bates et al., 2015). Session (hypoxic vs normoxic), domain (gains vs losses), and probability ratio were included as fixed effects, and participants as a random effect, with an unstructured covariance matrix. Session and domain were included in the model as categorical (dichotomous) predictors, with probability ratio as a continuous, centered predictor. Significant interactions between factors were analyzed by means of post-hoc tests, adjusting *p*-values for multiple comparisons with Bonferroni correction. The main statistics of the GLMM analysis are reported in Table 1. The model values of AIC and BIC were 12,746 and 12,812, respectively; log-likelihood value was -6364.4, *R* ^2^ marginal 0.03 and *R* ^2^ conditional 0.24.

**TABLE 1:** Main results of GLMM analysis performed on participants’ responses in the decision-making task.

Variables		Estimate	SE	exp(*B*)	95% Confidence Interval		z	*p*
					Lower	Upper		
Intercept		−0.336	0.191	0.714	0.491	1.038	−1.77	0.078
Session	Hypoxia—Normoxia	0.166	0.043	1.180	1.085	1.284	3.85	< .001
Domain	Gain - Loss	−0.395	0.043	0.674	0.619	0.734	−9.17	< .001
Probability ratio		0.025	0.022	1.025	0.982	1.069	1.13	0.260
Session ✻ Domain	Hypoxia—Normoxia ✻ Gain—Loss	−0.451	0.086	0.637	0.538	0.754	−5.24	< .001
Session ✻ Domain ✻ Probability ratio	Normoxia ✻ Loss ✻ Probability ratio	−0.266	0.033	0.766	0.719	0.817	−8.11	< .001
	Hypoxia ✻ Loss ✻ Probability ratio	−0.139	0.031	0.870	0.819	0.924	−4.53	< .001
	Normoxia ✻ Gain ✻ Probability ratio	0.062	0.031	1.064	1.001	1.130	2.03	0.043
	Hypoxia ✻ Gain ✻ Probability ratio	NaN	NaN	NaN	NaN	NaN	NaN	NaN

**FIGURE 2:**
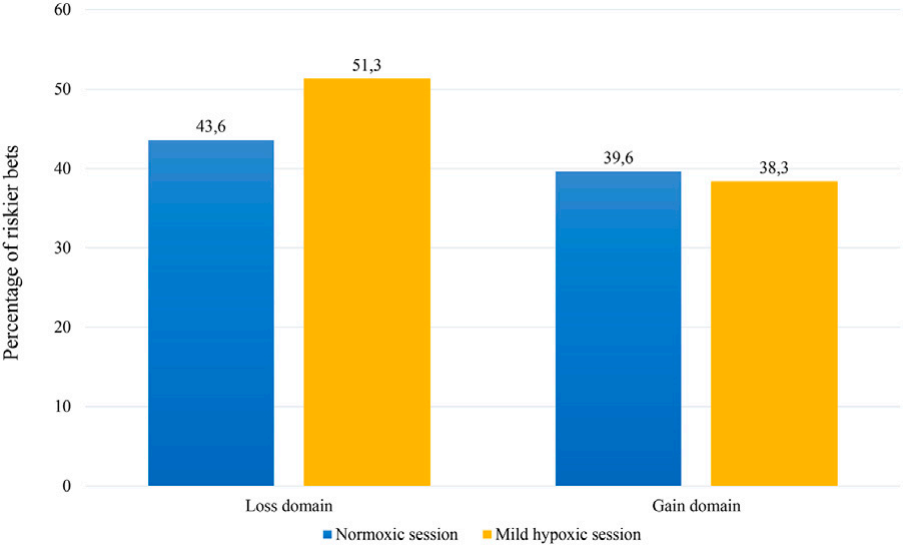
Probability of choosing the riskier bet as a function of probability ratio by session (normoxic and mild hypoxic) and domain (loss and gain).

In line with previous findings, participants’ choices were affected by the oxygen manipulation (Figure 2). Overall, individuals chose the riskier bet more often in the mild hypoxic than in the normoxic session (*p* < .001). This main effect was qualified by a significant interaction between session and domain (*p* < .001). For losses participants chose the riskier bets more often in the mild hypoxic than normoxic session (exp(*B*) = 0.676, *p* < .001), but for gains session had no impact on participants’ choices (exp(*B*) = 1.062, *p* = .281).

**FIGURE 3:**
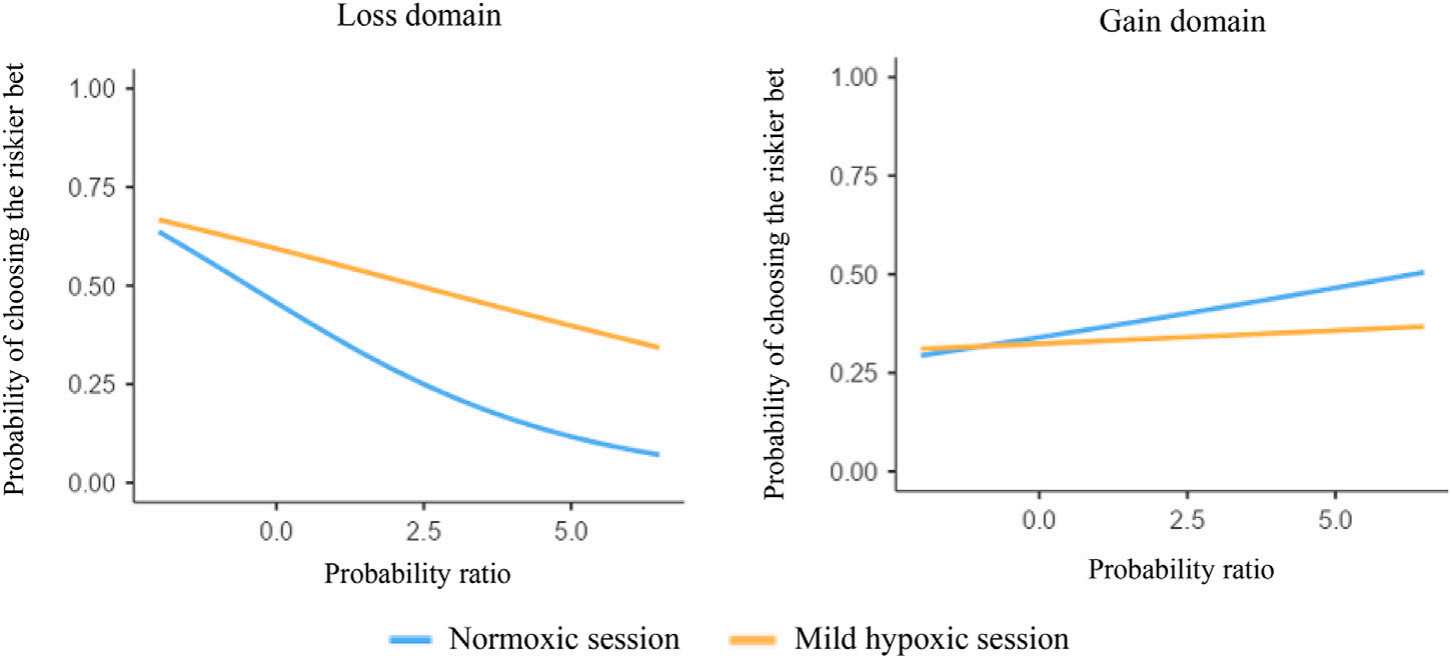
Percentage of choices of the riskier bet in the two sessions (normoxic and mild hypoxic) and for the two domains (losses and gains).

Importantly, we found a significant three-way interaction between session, domain, and probability ratio (*p* < .001). For losses (Figure 3, left panel), the increased preference for the riskier bet under mild hypoxia was positively associated with probability ratio. Faced with two potential losses, as the probability ratio increased, individuals in the mild hypoxic session chose the riskier bet *more often* than in the normoxic session (exp(*B*) = 1.235, 95% *CI*, 1.139–1.338, *p* < .001). For gains (Figure 3, right panel), although choices did not significantly differ between experimental sessions, as the probability ratio increased, individuals in the mild hypoxic session chose the riskier bet *less often* than in the normoxic session (exp(*B*) = 0.927, 95% *CI*, 0.868–0.990, *p* = .023).

To better qualify the main findings, further analyses were carried out (supplementary results can be found at https://osf.io/x3j2q/).

First, the main GLMM analysis was repeated by including participants’ assumed awareness concerning the ongoing oxygen condition (incorrect vs correct) as covariate. Main results remained unchanged, although participants’ capacity to correctly guess the experimental condition was, per se, a significant predictor of riskier choices (exp(*B*) = 1.382, 95% *CI*, 1.222–1.563, *p* < .001). Interestingly, analysis of the significant interaction effect between session and awareness revealed that the oxygen depletion significantly increased the number of riskier choices when participants’ guesses were incorrect (that is when participants had no correct insights about the ongoing oxygen condition; exp(*B*) = 0.666, *p* < .001) but not when their guesses were correct (exp(*B*) = 0.952, *p* > .05). More specifically, post-hoc comparisons aimed at explaining the significant three-way interaction among session, domain and awareness showed a significant increase of riskier choices in the mild hypoxic compared to the normoxic session, but only in the loss domain and only when participants were unable to recognize the condition they were in (exp(*B*) = 0.417, *p* < .001).

Second, in order to rule out the possibility that the observed results were due to participants’ perception of being in a specific oxygen condition (regardless of the actual condition), the main binomial GLMM analysis on decisions was repeated by replacing the actual condition (experimental session) with participants’ perception of the experimental condition, leaving the rest unchanged. Results showed that participants’ perception was not a significant predictor of their decisions (exp(*B*) = 0.923, 95% *CI*, 0.822–1.036, *p* = .175). Furthermore, the main GLMM analysis was repeated by including participants’ perception of the experimental condition as covariate. Again, the main pattern of results did not change, as the proportion of riskier choices still varied as a function of the experimental session (exp(*B*) = 1.255, 95% *CI*, 1.147–1.372, *p* < .001) even controlling for participants’ perception of the experimental session.

## 4 Discussion

The present results are in line with previous research (Pighin et al., 2012; Pighin et al., 2020), in that they show that mild hypoxia, which remains undetected by participants, significantly alters risk-taking behavior. When faced with potential losses, participants showed a higher preference for the riskier bet under mild hypoxia than normoxia, but the oxygen depletion had no effect on gains.

Importantly, the results also show that mild hypoxia moderates the effect of probability ratio on risky choices. A mild oxygen depletion increased the preference for the riskier bet in the loss domain, especially for pairs of bets posing very different probabilities and payoffs (i.e., pairs with a high probability ratio; e.g., 90% chance of losing €3 vs 10% chance of losing €27). In the gain domain, instead, mild hypoxia decreased the preference for the riskier bet, especially for pairs of bets posing very different probabilities and payoffs (i.e., pairs with a high probability ratio; e.g., 90% chance of winning €3 vs 10% chance of winning €27). A straightforward interpretation of this result is that, under mild hypoxia, individuals are more prone to expose themselves to substantial but unlikely losses and less attracted by substantial but unlikely gains.

Therefore, a mild oxygen depletion might alter the relative focus that individuals place on a bet’s probability and payoff. It has been argued that individuals who prefer riskier over safer bets focus primarily on payoff rather than probability (Slovic and Lichtenstein, 1968). For example, the preference for a 1% chance of winning 90 euros rather than a 90% chance of winning 1 euro is believed to be driven by the hope of a large gain (Kahneman, 2012). The finding that, for pairs of bets with a high probability ratio, individuals prefer riskier bets for gains less often in a mildly hypoxic than a normoxic environment may imply that oxygen depletion promotes the focus on probabilities and decreases the focus on payoffs.

Another interpretation of the present results can be traced to the probability weighting function proposed by prospect theory (Kahneman and Tversky, 1979), one of the most comprehensive theories of decision making under uncertainty. The theory argues that individuals incorporate in their decision a transformation of probability that considers the psychophysical notion of diminishing sensitivity, where the impact of any change in probability diminishes as the distance from the natural borders (certainty and impossibility) increases. This feature is captured by an inverse S-shaped weighting function and accounts for individuals’ tendency to overweight (*w* [*p*]>*p*) small probabilities and underweight (*w* [*p*]<*p*) moderate to high probabilities. The shape of the probability weighting function explains the certainty effect and the possibility effect. The certainty effect is observed when medium-high probability prospects are at issue, and people show risk-seeking for losses by preferring a potential loss over a sure loss (e.g., an 80% chance of losing €12.5 to a 100% chance of losing €10), and risk aversion for gains by preferring a sure gain over a potential gain (e.g., an 100% chance of receiving €10 to an 80% chance of receiving €12.5). The possibility effect, conversely, is observed with low probability prospects and involves the opposite trend: risk aversion for losses due to the overweighting of the small probability of a large loss (e.g., people prefer a 100% chance of losing €1 over a 5% chance of losing €20), but risk seeking for gains due to the overweighting of the small probability of a large gain (e.g., people prefer a 5% chance of receiving €20 over a 100% chance of receiving €1).

In the current study, the pairs of bets with a high probability ratio included a high-probability low-payoff bet and a low-probability high-payoff bet, a situation that resembles one in which the possibility effect is observed. Coherently with what is predicted by the possibility effect, as the probability ratio increased, participants in our study showed a lower tendency to choose the riskier bet in the domain of losses and a higher tendency to choose the riskier bet in the domain of gains. Interestingly, however, these tendencies were less pronounced under mild hypoxia than under normoxia, possibly suggesting that mild hypoxia attenuates the overweighting of small probabilities. Mild hypoxia, therefore, appears to inhibit the tendency to become more risk averse when facing the small probability of incurring a substantial loss, as well as the tendency to become more risk seeking when facing the small probability of obtaining a substantial gain.

Due to the way our stimuli were designed (i.e., the bets in each pair were matched in expected value), our results are mute as to whether mild hypoxia affects rational choices normatively. Moreover, as mentioned above, since the expected value of the bets was held constant in each pair, varying the probability ratio necessitated a symmetrical variation in the payoff ratio. This has the benefit of keeping the two bets comparable in terms of expected monetary value, but it has the drawback of ruling out any disentangling of the effects of mild hypoxia on how individuals perceive and treat probability and payoffs (winnings or losses) separately. Future studies could address this limitation by employing stimuli in which probability and payoff do not systematically covary. Nevertheless, the present investigation sheds light on the intricate relationship between human decision making and stress, providing useful novel insights for translational applications and into real-life context. Our results suggest that a stressor can exert an influence on risk propensity while remaining undetected. Even if we must be cautious in interpreting the effect of participants’ awareness of the oxygen condition (since this was not experimentally manipulated but simply reflected participants’ perception of the ongoing situation), the observed results suggest that mild hypoxia has the greatest effect on choices involving possible losses, especially for those participants who were unable to recognize the condition they are in. Thus, cognitive appraisal of a situation as stressful might play a role, though it seems not a crucial one, in determining the observed change in behavior. This is a distinguishing feature between the results obtained under mild hypoxia and under other (aware) stressful conditions, which might account, at least partially, for the inconsistencies between them (Porcelli and Delgado, 2009; Clark et al., 2012; Starcke and Brand, 2012, 2016; Pabst, Brand and Wolf, 2013; Pighin et al., 2015). Future studies should build on the present results to further investigate the ties between awareness and the effect of stress on decision making. Unraveling the links between these variables could be of great importance, especially in differentiating the role of pure physiological stressors from that of psychological stressors in exerting stress-regulative, top-down processes that ultimately impact behavior.

## Data Availability

The datasets presented in this study can be found here: https://osf.io/x3j2q/.
